# The diversity, evolution and ecology of *Salmonella* in venomous snakes

**DOI:** 10.1371/journal.pntd.0007169

**Published:** 2019-06-04

**Authors:** Caisey V. Pulford, Nicolas Wenner, Martha L. Redway, Ella V. Rodwell, Hermione J. Webster, Roberta Escudero, Carsten Kröger, Rocío Canals, Will Rowe, Javier Lopez, Neil Hall, Paul D. Rowley, Dorina Timofte, Robert A. Harrison, Kate S. Baker, Jay C. D. Hinton

**Affiliations:** 1 Department of Functional and Comparative Genomics, Institute of Integrative Biology, University of Liverpool, Liverpool, United Kingdom; 2 Animal Health Department, Chester Zoo, Cheshire, United Kingdom; 3 Earlham Institute, Norwich Research Park, Norwich, United Kingdom; 4 School of Biological Sciences, University of East Anglia, Norwich, United Kingdom; 5 Centre for Snakebite Research and Interventions, Liverpool School of Tropical Medicine, Liverpool, United Kingdom; 6 Institute of Veterinary Science, University of Liverpool, Leahurst Campus, Cheshire, United Kingdom; 7 Institute of Infection and Global Health, University of Liverpool, Liverpool, United Kingdom; Johns Hopkins Bloomberg School of Public Health, UNITED STATES

## Abstract

**Background:**

Reptile-associated *Salmonella* bacteria are a major, but often neglected cause of both gastrointestinal and bloodstream infection in humans globally. The diversity of *Salmonella enterica* has not yet been determined in venomous snakes, however other ectothermic animals have been reported to carry a broad range of *Salmonella* bacteria. We investigated the prevalence and diversity of *Salmonella* in a collection of venomous snakes and non-venomous reptiles.

**Methodology/Principle findings:**

We used a combination of selective enrichment techniques to establish a unique dataset of reptilian isolates to study *Salmonella enterica* species-level evolution and ecology and used whole-genome sequencing to investigate the relatedness of phylogenetic groups. We observed that 91% of venomous snakes carried *Salmonella*, and found that a diverse range of serovars (*n* = 58) were carried by reptiles. The *Salmonella* serovars belonged to four of the six *Salmonella enterica* subspecies: *diarizonae*, *enterica*, *houtanae* and *salamae*. Subspecies *enterica* isolates were distributed among two distinct phylogenetic clusters, previously described as clade A (52%) and clade B (48%). We identified metabolic differences between *S*. *diarizonae*, *S*. *enterica* clade A and clade B involving growth on lactose, tartaric acid, dulcitol, *myo*-inositol and allantoin.

**Significance:**

We present the first whole genome-based comparative study of the *Salmonella* bacteria that colonise venomous and non-venomous reptiles and shed new light on *Salmonella* evolution. Venomous snakes examined in this study carried a broad range of *Salmonella*, including serovars which have been associated with disease in humans such as *S*. Enteritidis. The findings raise the possibility that venomous snakes could be a reservoir for *Salmonella* serovars associated with human salmonellosis.

## Author summary

*Salmonella enterica* is a remarkable bacterial species that causes Neglected Tropical Diseases globally. The burden of disease is greatest in some of the most poverty-afflicted regions of Africa, where salmonellosis frequently causes bloodstream infection with fatal consequences. The bacteria have the ability to colonise the gastrointestinal tract of a wide range of animals including reptiles. Direct or indirect contact between reptiles and humans can cause salmonellosis. In this study, we determined the prevalence and diversity of *Salmonella* in a collection of African venomous snakes for the first time. We showed that the majority of venomous snakes (91%) in our study carry *Salmonella*, and used bacterial genomics to assign two thirds of isolates to the *S*. *enterica* subspecies *enterica* which is associated with human salmonellosis. We identified two evolutionary groups of *S*. *enterica* subspecies *enterica* that display distinct metabolic profiles with infection-relevant carbon sources. Our findings could have a broad significance because venomous snakes can move freely around human dwellings in tropical regions of the world such as Africa, and could potentially shed contaminated faecal matter onto surfaces and into water supplies.

## Introduction

*Salmonella* is a clinically relevant bacterial pathogen that poses a significant burden upon public health worldwide [1–4]. Two groups of *Salmonella* serovars have clinical relevance with distinct host-specificity and disease manifestations. Typhoidal *Salmonella* is restricted to human hosts and presents as a systemic infection, resulting in an estimated 223,000 fatalities per annum [5]. In contrast, nontyphoidal *Salmonella* typically manifests as a self-limiting gastrointestinal disease in otherwise healthy individuals around the world, causing an annual global disease burden of 93.8 million cases and 155,000 deaths [3]. Over the past two decades, an invasive form of nontyphoidal *Salmonella* (iNTS) has emerged as the most prevalent bacterial species to be isolated from the bloodstream of patients in sub-Saharan Africa [6]. Over 3.4 million cases and 680,000 deaths are estimated to occur worldwide each year as a result of iNTS [4].

The *Salmonella* genus contains two species; *S*. *bongori* and *S*. *enterica*. *S*. *enterica* is further divided into six subspecies; *enterica* (I), *salamae* (II), *arizonae* (IIIa), *diarizonae* (IIIb), *houtanae* (IV) and *indica* (VI) [7]. The subspecies are classified into approximately 2 600 serovars which are ecologically, phenotypically and genetically diverse [8]. Serovars which belong to *S*. *enterica* subspecies *enterica* cluster phylogenetically into two predominant clades (A and B) [9–11]. Here, we use the term *Salmonella* to refer to the *S*. *enterica* species and the *S*. *enterica* designation to refer to the *S*. *enterica* subspecies *enterica* alone, unless stated otherwise.

Biochemical properties such as carbon utilisation and anaerobic metabolism are often serovar-specific [12]. The ability of *Salmonella* to grow in a wide range of conditions reflects the adaptation of the bacteria to survive in the environment or in different hosts, as demonstrated by a recent study focused on genome-scale metabolic models for 410 *Salmonella* isolates spanning 64 serovars in 530 different growth conditions [13].

At the genus level, *Salmonella* has a broad host-range whilst individual serovars differ in host-specificity [14]. The majority of *Salmonella* infections in humans (99%) are caused by a small number of serovars belonging to the *S*. *enterica* subspecies [15]. Serovars which belong to non-*enterica* subspecies are associated with carriage in ectothermic animals such as reptiles and amphibians, but are rarely found in humans [14,16–18]. Carriage rates of non-*enterica* serovars in reptiles can be high. A study focused on snakes in a pet shop found that 81% of animals were carrying *S*. *diarizonae* [18]. Previous studies demonstrating the diverse range of *Salmonella* subspecies that colonise various reptilian species in different countries are summarised in Table 1.

**Table 1:** The distribution of *Salmonella* subspecies in non-venomous reptiles.

Citation	Country of study	Reptile information	Proportion of reptiles carrying *Salmonella* in the study	Subspecies Composition
Piasecki *et al*., 2014 [19]	Poland	Snakes, Lizards, Chelonians	122 of 374 (32.6%)	59% *enterica*, 16% *salamae* or *houtanae*, 3.5% *diarizonae* or *indica*
Lukac *et al*., 2015 [20]	Croatia	Snakes, Lizards, Chelonians	26 of 200 (13.0%)	34.6% *enterica*, 23.1% *houtanae*, 23.1% *arizonae*, 15.4% *diarizonae*, 2.8% *salamae*
Nakadai *et al*., 2005 [21]	Japan	Snakes, Lizards, Turtles	83 of 112 (74.1%)	62.5% *enterica*, 37.5% *salamae*, *arizonae*, *diarizonae* or *houtanae*
Geue and Löschener, 2002 [22]	Germany and Austria	Reptiles	86 of 159 (54.1%)	52.8% *enterica*, 34.5% *diarizonae*, 3.4% *salamae*, 6.9% *arizonae*, 2.3% *houtanae*
Sá and Solari, 2001 [23]	Brazil	Brazilian and Imported Pet Snakes, Lizards and Chelonians	38 of 97 (39.1%)	44.7% *enterica*, 10.5% *salamae*, 5.2% *arizonae*, 21% *diarizonae*, 18.5% *houtanae*
Perderson *et al*., 2009 [24]	Denmark	Captive reptiles	Not discussed	65% *enterica*, 12% *diarizonae*, 11% *houtanae*, 6% *salamae*, 6% *arizonae*
Schröter *et al*., 2004 [18]	Germany	Captive reptiles	13 of 16 (81.3%)	100% *diarizonae*

Reptiles represent a significant reservoir for serovars of *Salmonella* that are associated with human disease. Over 60% of captive-bred reptiles between 1995 and 2006 in Denmark were reported to carry *S*. *enterica* subspecies *enterica* serovars [24]. About 6% of human salmonellosis cases were contracted from reptiles in the USA [25], and in South West England, 27.4% of *Salmonella* cases in children under five years old were linked to reptile exposure [26]. The latter study demonstrated that reptile-derived salmonellosis was more likely to cause bloodstream infection in humans than non-reptile-derived *Salmonella* [26]. Reptile-associated *Salmonella* is therefore considered to be a global threat to public health [27].

The majority of reptile-associated salmonellosis cases reported in humans are caused by *Salmonella* from non-venomous reptiles [27], probably because these animals are frequently kept as pets. Therefore, non-venomous reptiles have been the focus of numerous studies whilst the prevalence and diversity of *Salmonella* in venomous snakes has remained unknown. The recent inclusion of snakebite as a neglected tropical disease demonstrates that these reptiles frequently interact with humans in tropical and sub-tropical countries. The proximity of venomous snakes to humans may lead to contaminated faecal matter being shed on the surfaces and in water sources used for human homes and to irrigate salad crops [28–30]. Research to improve snakebite treatment at the Liverpool School of Tropical Medicine (LSTM) has resulted in the creation of the most extensive collection of venomous snakes in the UK (195). The LSTM herpetarium houses venomous snakes from a diverse range of species and geographical origins, representing an ideal source of samples to assess *Salmonella* in this under-studied group of reptiles.

The aims of this study were three-fold. Firstly, to determine the period prevalence of *Salmonella* in a collection of captive venomous snakes and investigate whether this group of reptiles could act as reservoirs for human salmonellosis. Secondly, to assess the serological and phylogenetic diversity of *Salmonella* amongst reptiles. Thirdly, to use the diversity of reptile-associated *Salmonella* to determine clade-specific differences that could reflect adaptation to survival in the environment or to different hosts. Here, we present the first whole genome-based comparative study of the *Salmonella* bacteria that colonise venomous and non-venomous reptiles.

## Methods

### Source of *Salmonella* isolates

The *Salmonella* isolates were derived from faecal samples from two collections of reptiles. One hundred and six faecal samples were collected from venomous snakes at LSTM from May 2015 to January 2017, with an emphasis on snakes originating from Africa (S1 Table), and investigated for the presence of *Salmonella*. All venomous snakes were housed in individual enclosures and fed with frozen mice. Sixty-nine of the samples (71%) were sourced from wild-caught snakes originating from: Togo, Nigeria, Cameroon, Egypt, Tanzania, Kenya, South Africa, and Uganda. A further 28 *Salmonella* isolates (29%) came from venomous snakes bred in captivity. The LSTM herpetarium is a UK Home Office licensed and inspected animal holding facility. A second collection of 27 *Salmonella* isolates from non-venomous reptiles and 1 *Salmonella* isolate from a venomous reptile were sourced from the veterinary diagnostics laboratory based at the University of Liverpool’s Leahurst campus (reptilian species described in S1 Table). These isolates were collected from June 2011 to July 2016 from specimens submitted as part of *Salmonella* surveillance for import/export, in addition to veterinary faecal samples and tissues from post mortem investigations. The provenance of the isolates is described in S1 Table. The majority of the non-venomous reptiles were sourced from a zoological collection, however two animals were privately owned and three were sourced from the Royal Society for the Prevention of Cruelty to Animals (RSPCA). The LSTM isolates are henceforth referred to as venomous snake isolates and the Leahurst isolates are referred to as non-venomous reptile isolates unless otherwise stated.

### Isolation of *Salmonella*

All media were prepared and used in accordance with the manufacturer’s guidelines unless otherwise stated. *Salmonella* was isolated using a modified version of the protocol described in the national Standard Operating Procedure for detection of *Salmonella* issued by Public Health England [31].

Faecal droppings were collected from reptiles and stored in 15 mL plastic centrifuge tubes at 4°C. Two different methods were used for the enrichment of *Salmonella* from faecal samples due to reagent availability at the time of isolation. S1 Table provides information on isolate specific methods. In enrichment method 1, faecal samples were added to 10 mL of buffered peptone water (Fluka Analytical, UK, 08105-500G-F) and incubated overnight at 37°C with shaking at 220 rpm. Following overnight incubation, 100 μL of the faeces mixture was added to 10 mL of Selenite Broth (19 g/L Selenite Broth Base, Merck, UK, 70153-500G and 4 g/L Sodium Hydrogen Selenite, Merck 1.06340-50G) and incubated overnight at 37°C with shaking at 220 rpm. In enrichment method 2, faecal samples were added to 10 mL of Buffered Peptone Water (Fluka Analytical, 08105-500G-F) supplemented with 10 μg/mL Novobiocin (Merck, N1628), and incubated overnight at 37°C with shaking at 220 rpm. Following overnight incubation, 100 μL of the faeces mixture was added to 10 mL of Rappaport-Vassilliadis Medium (Lab M, UK, LAB086) and incubated for 24 hours at 42°C with shaking at 220 rpm.

Following enrichment, 10 μL of overnight broth was spread onto Xylose Lysine Deoxycholate (XLD) (Oxoid, UK, CM0469) agar plates which were incubated overnight at 37°C. Putative *Salmonella* colonies were selected by black appearance on XLD plates and confirmed by pink and white colony formation on Brilliant Green Agar (Merck, 70134-500G) supplemented with 0.35 g/L Mandelic Acid (Merck, M2101) and 1 g/L Sodium Sulfacetamide (Merck, S8647).

To identify *S*. *enterica* species, colony PCR of the *Salmonella* specific *ttr* locus, which is required for tetrathionate respiration [32], was performed. PCR reagents included MyTaq Red Mix 1x (Bioline, UK, BIO-25043), *ttr-4* reverse primer (5'-AGCTCAGACCAAAAGTGACCATC-3') and *ttr-6* forward primer (5'-CTCACCAGGAGATTACAACATGG-3') on colonies suspected to be *Salmonella*. PCR reaction conditions were as follows: 95°C 2 min, 35 x (95°C 15 s, 60°C 30 s, 72°C 10 s), 72°C 5 min. PCR products were visualised using agarose gel (3.5%) (Bioline, BIO-41025) electrophoresis in TAE buffer. Midori Green DNA stain (3 μL/100 mL) (Nippon Genetics, Germany, MG 04) was used to visualise DNA bands under UV light. Throughout the isolation procedure, *S*. *enterica* serovar Typhimurium (*S*. Typhimurium) strain LT2 [33,34] was used as a positive control, and *Escherichia coli* MG1655 [35] was used as a negative control (S1 Table).

### Whole-genome sequencing of *Salmonella* from venomous and non-venomous reptiles

All non-venomous reptile isolates, one venomous reptile isolate and 87 of 97 venomous snake isolates were sent for whole-genome sequencing. Isolates were sent to either MicrobesNG, UK or the Earlham Institute, UK for whole-genome sequencing on the Illumina HiSeq platform (Illumina, California, USA). Isolates which were sequenced by MicrobesNG were prepared for sequencing in accordance with the company’s preparation protocol for single colony-derived bacterial cultures (http://www.microbesng.uk).

Isolates which were sequenced by the Earlham Institute were prepared by inoculating a single colony of *Salmonella* into a FluidX 2D Sequencing Tube (FluidX Ltd, UK) containing 100 μL of Lysogeny Broth (LB, Lennox) and incubating overnight at 37°C, with shaking at 220 rpm. LB was made using 10 g/L Bacto Tryptone (BD Biosciences, UK, 211705), 5 g/L Bacto Yeast Extract (BD, 212750) and 5 g/L Sodium Chloride (Merck, S3014-1kg). Following overnight growth, the FluidX 2D Tubes were placed in a 95°C oven for 20 minutes to heat-kill the isolates.

DNA extractions and Illumina library preparations were conducted using automated robots at MicrobesNG or the Earlham Institute. At the Earlham Institute, the Illumina Nextera XT DNA Library Prep Kit (Illumina, FC-131-1096) was used for library preparation. High throughput sequencing was performed using an Illumina HiSeq 4000 sequencing machine to generate 150 bp paired-end reads. Sequencing was multiplexed with 768 unique barcode combinations per sequencing lane. The insert size was approximately 180 bp, and the median depth of coverage was 30x.

At MicrobesNG (https://microbesng.uk), genomic DNA libraries were prepared using the Nextera XT Library Prep Kit (Illumina, FC-131-1096) with two nanograms of DNA used as input and double the elongation time that was described by the manufacturer. Libraries were sequenced on the Illumina HiSeq 2500 using a 250 bp protocol.

### Obtaining contextual reference sequences

The *Salmonella In Silico* Typing Resource (SISTR) v1.0.2 was used for serovar prediction [36]. Enterobase [37] was used to assign a Multi Locus Sequence Type (MLST) to each isolate, based on sequence conservation of seven housekeeping genes [37]. Where available, reference isolates representing previously sequenced *Salmonella* isolates for all subspecies and serovars identified were included in the analysis. Reference sequence assemblies were downloaded from the National Center for Biotechnology Information (NCBI). Accession numbers are available in S2 Table.

### Quality control checks

Fastqc v0.11.5 (https://www.bioinformatics.babraham.ac.uk/projects/fastqc/) and multiqc v1.0 (http://multiqc.info) were used to assess read quality. Kraken v0.10.5-beta [38] was run to ensure reads were free from contamination using the MiniKraken 8gb database and a *Salmonella* abundance cut-off of 70%. Trimmomatic v0.36 [39] was then used on the paired end reads to trim low-quality regions using a sliding widow of 4:200. ILLUMINACLIP was used to remove adapter sequences.

### Phylogenetics- core genome alignment tree

Genomes were assembled using SPADES v3.90 [40]. QUAST v4.6.3 [41] was used to assess the quality of assemblies, the results of which can be found in S3 Table. Assemblies which comprised of greater than 500 contiguous sequences were deemed too fragmented for downstream analysis. All assemblies which passed QC were annotated using Prokka v1.12 [42]. Roary v3.11.0 [43] was used to generate a core genome alignment. SNP-sites v2.3.3 [44] was used to extract SNPs. A maximum likelihood tree was built from the core genome SNP alignment of all isolates using RAxML-NG v0.4.1 BETA [45] with the general time reversible GTR model and gamma distribution for site specific variation and 100 bootstrap replicates to assess support. The tree was rooted using the *Salmonella* species *S*. *bongori*. Interactive Tree Of Life v4.2 [46] was used for tree visualisation. We confirmed that there was no bias in phylogenetic signal between the two different sequencing platforms used by assessing clustering patterns within the phylogenies. S1 Table contains details of the sequencing facility. Monophyletic clustering of isolates was used to assign subspecies to newly sequenced *Salmonella* from venomous and non-venomous reptilian hosts. The level of association between venom status and phylogenetic clade was determined using odds ratios and χ2 statistics using the OpenEpi website (http://www.openepi.com).

### Identification of clade-specific genomic regions

Genes involved in the utilisation of each carbon source were identified using KEGG [47] and relevant literature [9,32,48–59] (see S4 Table). Genes involved in the uptake of carbon sources were prioritised. Sequences were downloaded using the online tool SalComMac [60], which allows the download of fasta sequences of the genes in *S*. Typhimurium strain 4/74. In the case of *lac* genes, the sequences were taken from the *E*. *coli* reference sequence MG1655. The sequences can be found in S1 Text.

The software tool MEGABLAST v2.2.17 [61] was used to perform a BLAST search of genes in the reptile-derived genomes against a custom-made database of genes diagnostic of *Salmonella* Pathogenicity Islands and genes involved in carbon utilisation. To confirm all MEGABLAST results, the short reads were mapped against each gene using BWA v0.7.10 [62] and SAMtools v0.1.19 [63]. The resulting bam files were manually assessed for gene presence and absence using Integrative Genomics Viewer v2.4.15 [64]. The results were plotted against the maximum likelihood phylogeny using Interactive Tree Of Life v4.2 [46].

### Carbon source utilisation

Differential carbon source utilisation of 39 reptile-derived *Salmonella* isolates from *S*. *diarizonae*, *S*. *enterica* clade A and *S*. *enterica* clade B was assessed. Filter-sterilised carbon sugar solutions were added into M9 (Merck, M6030-1kg) agar at concentrations detailed in S5 Table. Isolated colonies were transferred from LB agar plates onto M9 carbon source plates using a sterile 48-pronged replica plate stamp and incubated at 37°C under aerobic conditions. An LB control plate was used to validate successful bacterial transfer and all experiments were performed in duplicate. If no growth was seen under aerobic conditions for a particular carbon source, the procedure was repeated under anaerobic conditions (approx. 0.35% oxygen) with 20 mM Trimethylamine *N-*oxide dihydrate (TMAO) (Merck, 92277) as a terminal electron acceptor. Anaerobic conditions were achieved by incubating plates in an anaerobic jar with 3x AnaeroGen 2.5 L sachets (Thermo Scientific, UK, AN0025A) to generate anaerobic gas. Oxygen levels were measured using SP-PSt7-NAU Sensor Spots and the Microx 4 oxygen detection system (PreSens, Regensburg, Germany). *Salmonella* growth was determined at 18, 90 and 162 hours in aerobic growth conditions and at 162 hours in anaerobic growth conditions. A sub-set of growth positive isolates were assessed for single colony formation to validate the results of the replica plating.

### Antimicrobial susceptibility testing of *Salmonella* isolated from venomous and non-venomous reptiles

Antimicrobial susceptibility was determined using a modified version of the European Committee on Antimicrobial Susceptibility Testing (EUCAST) disk diffusion method [65] using Mueller Hinton (Lab M, LAB039) agar plates and a DISKMASTER dispenser (Mast Group, UK, MDD64). Inhibition zone diameters were measured and compared to EUCAST zone diameter breakpoints for Enterobacteriaceae [66]. Isolates were first tested with six commonly used antibiotics (Ampicillin 10 μg, Chloramphenicol 30 μg, Nalidixic Acid 30 μg, Tetracycline 30 μg, Ceftriaxone 30 μg, and Trimethoprim/Sulfamethoxazole 25 μg) and were then tested with five additional antibiotics (Meropenem 10 μg, Gentamicin 10 μg, Amoxicillin/Clavulanic Acid 30 μg, Azithromycin 15 μg, and Ciprofloxacin 5 μg) if any resistance was seen to the primary antibiotics (all disks from Mast Group). If resistance was observed phenotypically, then the presence of antimicrobial resistance genes were investigated using the Resfinder software tool [67]. Antimicrobial resistance was defined as resistance to one antimicrobial to which isolates would normally be susceptible [68]. Multidrug resistance was defined as an isolate which showed resistance to three or more antimicrobials to which it would normally be susceptible [68].

## Results and discussion

### Similarities in *Salmonella* prevalence between captive venomous snakes and non-venomous reptiles

*Salmonella* carriage is well documented amongst reptiles (Table 1), however, to our knowledge no published study reports the incidence of *Salmonella* in venomous snakes. The period prevalence of *Salmonella* was assessed in a collection of 106 venomous snakes housed at the LSTM venom unit between May 2015 and January 2017. A remarkably high proportion (91%; 97/106) of the faecal samples contained *Salmonella* (S1 Table), which should be seen in the context of the significant carriage rate of *Salmonella* by other non-venomous reptiles described in the literature [27]. Variable rates of *Salmonella* carriage have been observed in collections of reptiles (Table 1), and the large proportion of venomous snakes carrying *Salmonella* in our study sits at the higher end of the reported spectrum. Our findings pose important public health considerations for individuals who work with venomous snakes housed in captivity, which may previously have been overlooked.

### The diversity of *Salmonella* highlights the possibility of local transmission events and the role of long-term shedding

**Fig 1:**
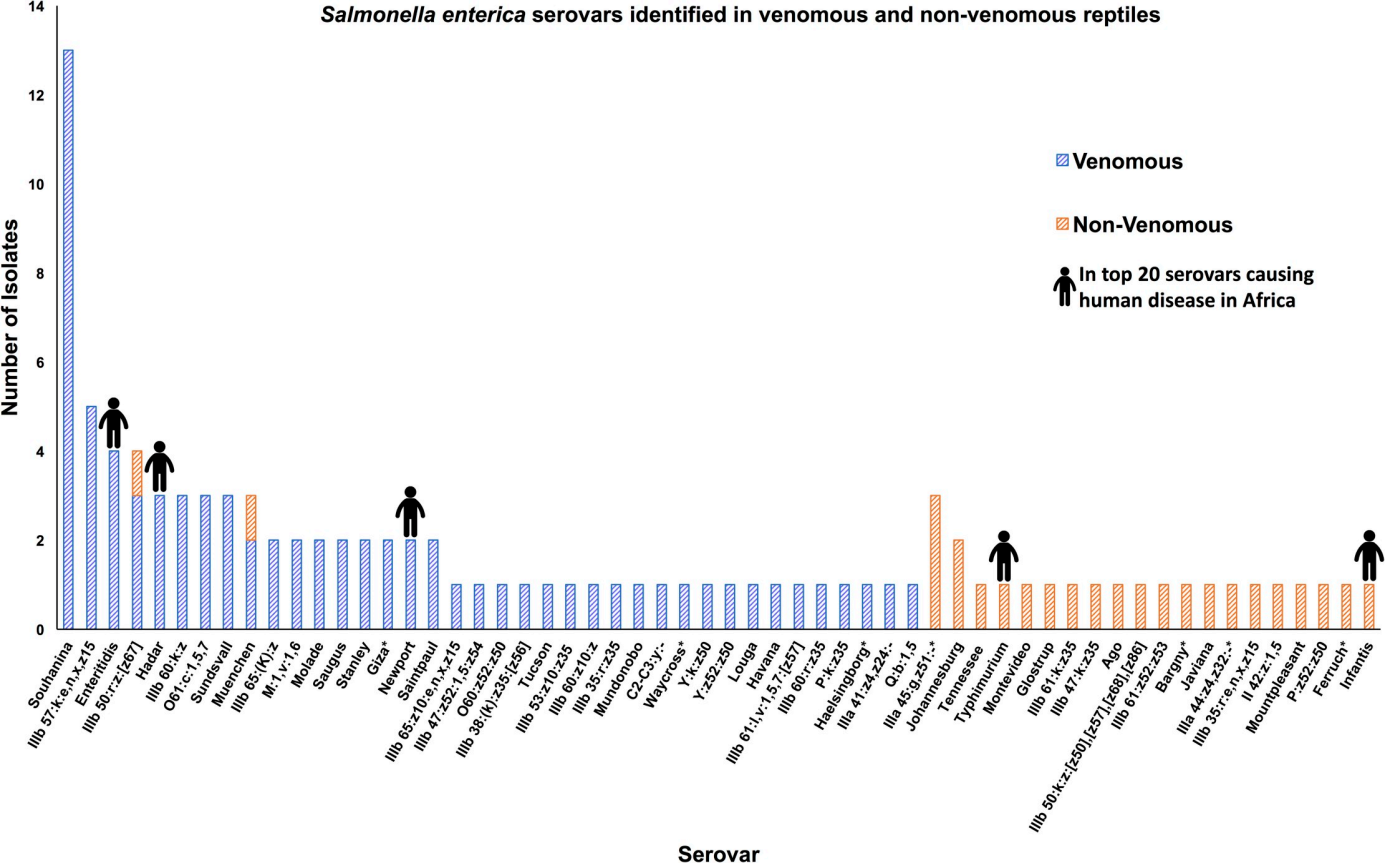
The distribution of the 58 *Salmonella enterica* serovars isolated from venomous and non-venomous reptiles. Each bar represents the total number of isolates which belonged to each serovar. Serovars containing isolates that had multiple serovar designations from SISTR are indicated with asterisks. Human pictographs are displayed on serovars which are amongst the top 20 isolated from humans in Africa. Data is based on the global monitoring of *Salmonella* serovar distribution from the WHO global foodborne infections network data [69].

To assess diversity, 87 venomous snake-derived *Salmonella* isolates, 27 non-venomous reptile-derived *Salmonella* isolates and one venomous reptile-derived *Salmonella* isolate were whole-genome sequenced. *In silico* serotyping revealed 58 different *Salmonella* serovars (Fig 1). A wide range of serovars was found in each of the venomous and non-venomous snake collections. Given that many of the serovars identified have a very broad host specificity, we suggest that the presence of particular serovars is not linked to the venom status of the reptile.

Similar levels of *Salmonella* carriage were seen in wild-caught and captive-bred reptiles. It is likely that the difference in serovar distribution reflects the sourcing of the reptiles from two independent housing facilities, and represents a limitation of our study. Nevertheless, an unprecedented level of *Salmonella* diversity was identified amongst both venomous and non-venomous reptiles. Following whole-genome sequencing, multi-locus sequence typing (MLST) was used for sub-serovar genetic characterisation of *Salmonella* [37]. In all cases, isolates falling within the same serovar had identical sequence types (S1 Table), reflecting the intra-serovar homogeneity of the *Salmonella* isolated in this study.

**Fig 2:**
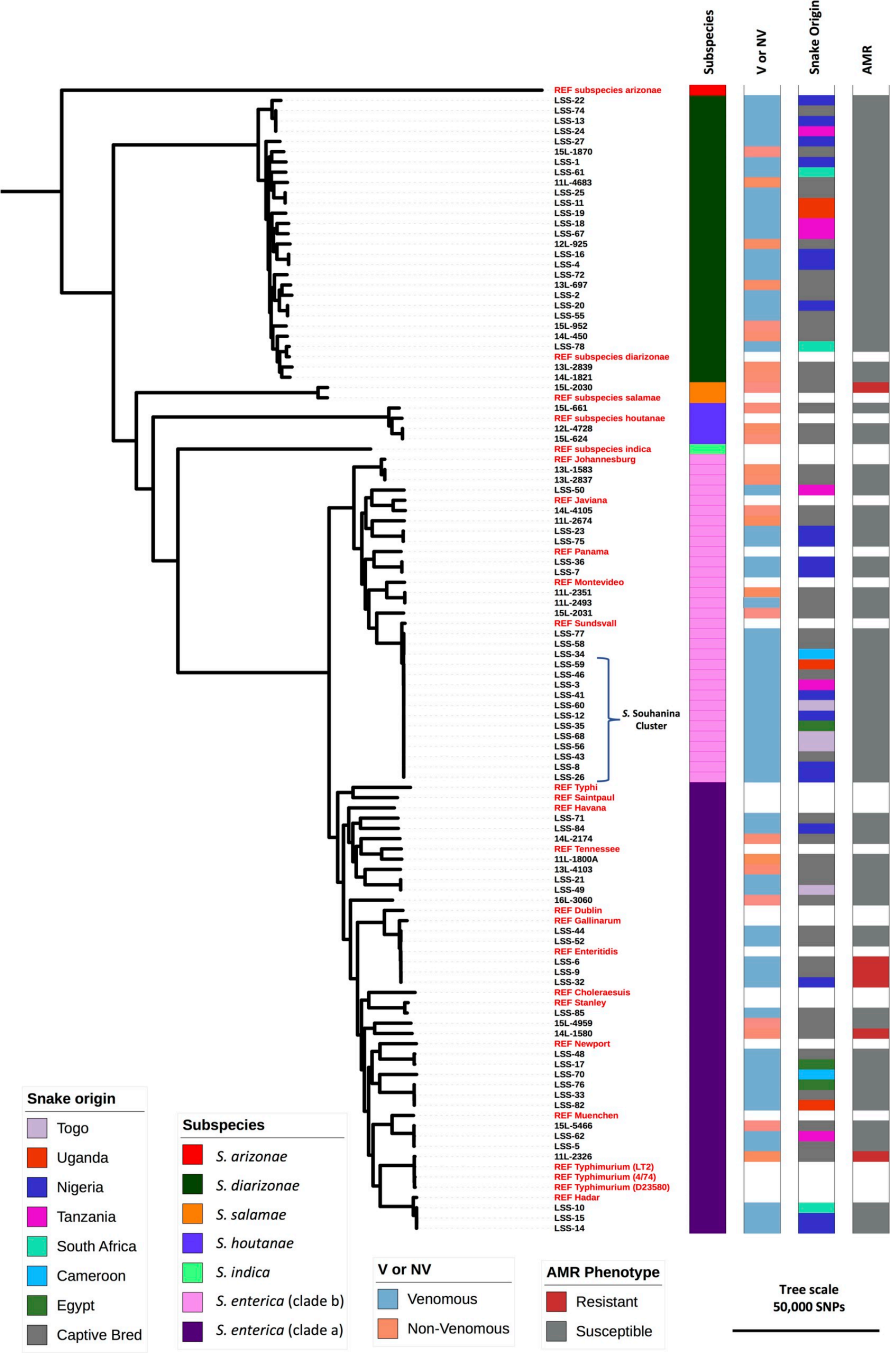
The diversity of *Salmonella* isolated from a collection of venomous snakes and non-venomous reptiles. Core genome maximum likelihood phylogenetic tree. The tree was rooted using *S*. *bongori* (S1 Fig). 25 contextual reference genomes representing previously sequenced isolates from each *Salmonella* subgroup are indicated in red. A cluster of *S*. Souhanina isolates which demonstrate a high level of genetic similarity are indicated. Colour strips showing metadata are as follows; Subspecies–Subspecies of isolate, Origin—The country of origin of the snake from which the isolate was taken, V or NV—Depicts whether the reptile host was venomous (V) or non-venomous (NV), AMR phenotype—Isolates shown in red were resistant to one or more antimicrobial agent. Metadata for the contextual reference genomes appear as white. Tree was visualised using ITOL (https://itol.embl.de).

The most common serovar to be identified amongst the venomous snake isolates was *S*. Souhanina (*n* = 12). All *S*. Souhanina isolates clustered locally on the phylogeny (Fig 2) falling within a 5 SNP cluster characteristic of a clonal expansion event [70]. Four of these isolates were found in captive-bred reptiles, whilst 11 isolates came from venomous snakes which originated in Cameroon, Uganda, Tanzania, Nigeria, Togo and Egypt. The close phylogenetic relationship between the *S*. Souhanina isolates that belong to the same MLST type (ST488) from imported animals with a range of origins and from captive animals suggests that local *Salmonella* transmission may be occurring. Local transmission of near-identical salmonellae could occur between snakes or as a result of a single contaminated food source such as frozen mice [71,72].

Although our data suggest that *S*. Souhanina was locally transmitted within the herpetarium, a single source of *Salmonella* would not explain the wide variety of serovars and MLST types carried by this collection of venomous snakes. Significant *Salmonella* diversity was reported in a study that involved 166 faecal samples from wild-caught reptiles in Spain, identifying 27 unique serovars [73]. An assessment of *Salmonella* diversity in wildlife in New South Wales, Australia identified 20 unique serovars amongst 60 wild-reptiles [74]. We speculate that the majority of the diversity of *Salmonella* identified here originated from wild-caught reptiles and reflect their varied habitats.

Underpinning our strategy for sampling *Salmonella* was the assumption that venomous snakes can carry and shed *Salmonella* for long periods of time. The longitudinal shedding of *Salmonella* has been reported in 12 captive non-venomous snakes from 7 different species. Over 10 consecutive weeks, 58% of the snakes shed *Salmonella* intermittently [75]. Chronic *Salmonella* carriage has been reported in many other animals, including laying hens which shed the bacteria continually for up to 10 weeks [76]. To assess the continuity of *Salmonella* shedding from venomous snakes in this study we collected three faecal samples from a Western Green Mamba from Togo over a three-month period between 31^st^ October 2016 and 31^st^ January 2017. All three faecal samples contained *Salmonella* which belonged to sequence type ST488, showing that individual snakes have the capacity to shed the same sequence type of *Salmonella* over a 90-day period in this study.

We propose that the majority of the reptile-derived *Salmonella* described in this study were acquired by reptiles prior to captivity, whilst some isolates were transmitted locally within the herpetarium.

### Venomous reptiles carry multidrug resistant *Salmonella* serovars of clinical relevance

Because the majority of venomous snakes examined in this study were of African origin or belonged to a species of snake native to the African continent (Fig 2), we compared the *Salmonella* serovars isolated from all reptiles in this study with those most frequently associated with human disease in Africa. The *Salmonella* serovar distribution has been reported by the WHO global foodborne infections network data bank based on data from quality assured laboratories in Cameroon, Senegal and Tunisia [69] (Fig 1). Eleven snake-derived isolates belonged to serovars commonly pathogenic in humans. This finding prompted us to determine the proportion of all venomous snakes and non-venomous reptiles that carried antimicrobial resistant *Salmonella* (Table 2). In *Salmonella* collected from venomous snakes, 4.1% of isolates (4/97) were resistant to at least one antimicrobial and two isolates were multidrug resistant (Table 2). Three resistant isolates from venomous snakes belonged to the serovar Enteritidis and were closely related to the global *S*. Enteritidis epidemic clade which causes human disease in Africa [77]. These findings demonstrate that venomous snakes are capable of carrying and shedding *Salmonella* that have the potential to cause disease in humans.

**Table 2:** Relating antimicrobial resistance to phenotype and genotype.

Isolate	Serovar^#^	Reptile	Scientific Name	Venom Status	Origin	AMR Resistance Phenotype*	Resistance Genes and Mutations	Antibiotic Family
LSS-6	Enteritidis	Monocled cobra	*Naja kaouthia*	Venomous	Captive Bred	Ampicillin	*blaTEM-1B*	Beta-lactam
						Nalidixic Acid	*gyrA* p.D87Y (GAC to TAC)	Quinolones
							*aph(3”)-1b*	Aminoglycoside
							*sul2*	Sulphonamide
LSS-9	Enteritidis	Monocled cobra	*Naja kaouthia*	Venomous	Captive Bred	Ampicillin	*blaTEM-1B*	Beta-lactam
						Chloramphenicol	catA2**	Phenicol
						Nalidixic Acid	*gyrA* p.D87G (GAC to GGC)	Quinolones
						Tetracycline	*tet(A)*	Tetracycline
						Cotrimoxazole	*sul2*	Sulphonamide
						Azithromycin	*aph(3”)-1b*, aph(6)-1d**	Aminoglycoside
LSS-28	Stanley	Malaysian Spitting Cobra	*Naja siamensis*	Venomous	Captive Bred	Ampicillin	*blaOXA-1*	Beta-lactam
						Tetracycline	tet(A)**	Tetracycline
							*aph(4)-la*, *aac(6’)lb-cr*, aac(3_1Va)**	Aminoglycoside
							*aac(6’)lb-cr*	Fluoroquinolone
							*sul2*, *sul1*	Sulphonamide
							catB3**	Phenicol
							*ARR-3*	Rifampicin
LSS-32	Enteritidis	Black-Necked Spitting Cobra	*Naja nigricollis*	Venomous	Nigeria	Ampicillin	*blaTEM-1B*	Beta-lactam
						Chloramphenicol	catA2**	Phenicol
						Nalidixic Acid	*gyrA* p.D87G (GAC to GGC)	Quinolones
						Tetracycline	*tet(A)*	Tetracycline
							*aph(3”)-1b*, aph(6)-1d**	Aminoglycoside
							*sul2*	Sulphonamide
11L-2326	Typhimurium	Python	*Pythonidae*	Non-Venomous	Captive Bred	Ampicillin	*blaCARB-2*	Beta-lactam
						Chloramphenicol	*floR*	Phenicol
						Nalidixic Acid	*gyrA* p.D87Y (GAC to TAC)	Quinolones
						Tetracycline	*tet(G)*	Tetracycline
						Cotrimoxazole	*sul1*	Sulfonamide
						Azithromycin	*aadA2*	Aminoglycoside
14L-1580	Unknown	Carpet Python	*Morelia spilota*	Non-Venomous	Captive Bred	Nalidixic Acid	*gyrA* p.D87G (GAC to GGC)	Quinolones
						Tetracycline	*tet(A)*	Tetracycline
15L-2030	II 42:z:1,5	Olive Tree Skink	*Dasia olivacea*	Non-Venomous	Captive Bred	Tetracycline	No resistance genes identified	

^#^ Serovar predicted from genome sequence with SISTR [36]

* Determined experimentally, as described in Materials and Methods

** Less than 100% sequence identity on ResFinder

NB: We have defined multidrug resistance as resistance to greater than three antibiotics.

### Phylogenetic diversity and molecular epidemiology of *Salmonella* in venomous snakes and non-venomous reptiles demonstrated for the first time

Here we have shown that venomous snakes can shed *Salmonella*. The vast diversity of *Salmonella* has long been acknowledged in the literature [37]. To study the diversity of reptile-associated *Salmonella* from an evolutionary perspective, we obtained 87 high quality whole-genome sequences for a phylogenetic comparison that involved 24 contextual *Salmonella* genomes (methods). The 87 genomes represented 60 isolates from venomous snakes, 26 *Salmonella* isolates from non-venomous reptiles and 1 *Salmonella* isolate from a venomous reptile. Following a comprehensive comparative genomic analysis, we identified a total of 405,231 core genome SNPs that differentiated the 87 isolates, and were used to infer a maximum likelihood phylogeny (Fig 2). SNPs are a valuable marker of genetic diversity [70], and the identification of hundreds of thousands of core-genome SNPs reflects a high level of genetic diversity among the reptile associated *Salmonella* isolates. The collection of reptile-derived *Salmonella* represented most of the known diversity of the *Salmonella* genus [7], spanning four of the six *Salmonella enterica* subspecies: *diarizonae*, *enterica*, *houtanae* and *salamae*. Reptile-derived *S*. *enterica* subspecies *enterica* isolates were approximately equally distributed among two distinct phylogenetic clusters, known as clade A (58%) and clade B (48%) [9–11] (Fig 2). No significant association was found between venom status and phylogenetic group (OR = 1.1, CI = 0.3–3.0, χ2 = 0.02, P = 0.4).

### Genotypic and phenotypic conservation of infection-relevant carbon source utilisation and virulence-associated genomic regions sheds new light on *Salmonella* ecology

The unique collection of diverse *Salmonella* isolates was used to determine the phenotypic and genotypic conservation of infection-relevant properties and genomic elements. Whilst the reptile-associated *Salmonella* belonged to five evolutionary groups, the majority of isolates were classified as *S*. *diarizonae* or *S*. *enterica*. The clustering of *S*. *enterica* into two clades (A and B) has previously been inferred phylogenetically based on the alignment of 92 core loci [9,10]. The biological significance of *S*. *enterica* clade A and clade B has been established as the two clades differ in host specificity, virulence-associated genes and metabolic properties such as carbon utilisation [11]. The genome sequences were used to expand upon pre-existing knowledge and determine phenotypic and genotypic conservation of metabolic and virulence factors across *S*. *diarizonae* and *S*. *enterica* (clades A and B).

Although the majority of *Salmonella* serovars of public health significance belong to clade A, certain clade B serovars such as *Salmonella* Panama have been associated with invasive disease [78,79]. The clade B *S*. *enterica* generally carry a combination of two *Salmonella* genomic islands. The *Salmonella* Pathogenicity Island-18 encodes an intracellularly expressed pore forming hemolysin *hlyE* and the cytolethal distending toxin islet which includes the gene *cdtB* [2,9]. It has been suggested that the two islands are associated with invasive disease, as previously they had only been identified in *S*. *enterica* serovar Typhi and Paratyphi A, which cause bloodstream infections [2,9]. The combination of *hlyE* and *cdtB* genes were present in all *S*. *diarizonae* and *S*. *enterica* clade B isolates in this study, but absent from all but one *S*. *enterica* clade A isolate (14L-2174). We propose that the significant proportion of reptiles which carried *S*. *enterica* clade B could partially explain the increased likelihood of reptile-associated salmonellosis involving invasive disease, compared to non-reptile-acquired salmonellosis [26].

**Fig 3:**
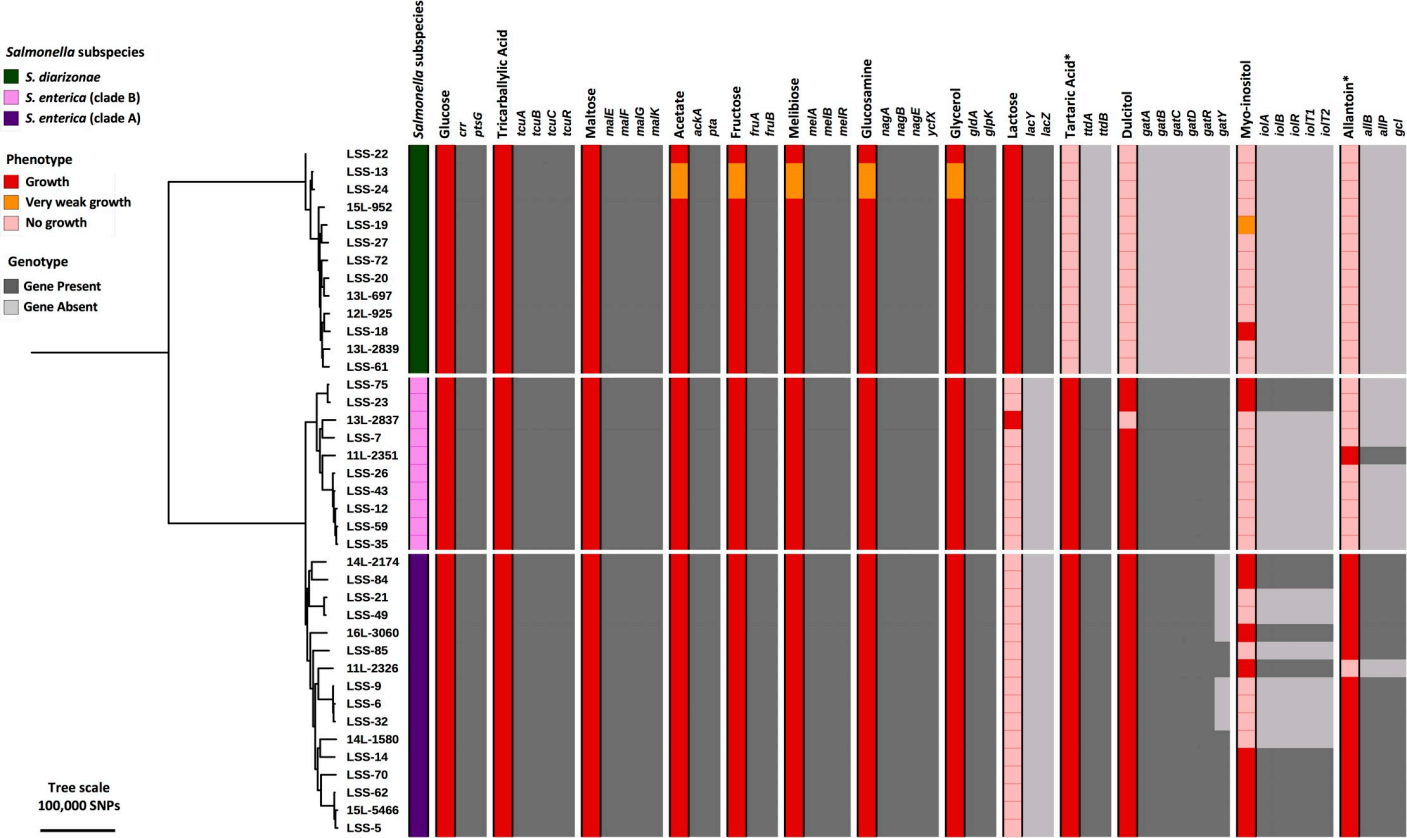
Phylogenetic context of carbon-source utilisation by reptile-derived-*Salmonella* isolates. Carbon source utilisation data were mapped against the core genome phylogenetic tree. The maximum likelihood tree includes all reptile-derived *Salmonella* isolates which were assessed for carbon utilisation and had high quality genome sequences (see S4 Table, S5 Table and S1 Text). Reference sequences for the majority of carbon utilisation and acquisition genes were taken from *S*. Typhimurium strain 4/74, and the *lac* gene sequences were from *E*. *coli* MG1655. Carbon sources which required anaerobic conditions are indicated with asterisk.

To assess metabolic differences that distinguish *S*. *enterica* clade A, *S*. *enterica* clade B and *S*. *diarizonae*, we phenotypically screened 39 reptile isolates for the ability to catabolise a number of infection-relevant carbon sources [48,77,80,81] (S4 Table and S5 Table). A summary of the results for phenotypic carbon utilisation and the presence of genes associated with the cognate metabolic pathway is shown in Fig 3.

In general, the genotype accurately reflected phenotype in terms of carbon source utilisation; however, this was not always the case (Fig 3). Discrepancies between phenotypic growth and genotype suggests that mechanisms of *Salmonella* metabolism remain to be elucidated. For example, *S*. *diarizonae* isolate LSS-18 grew well on *myo*-inositol as a sole carbon source (Fig 3) but showed zero percent homology with any of the *iol* genes from the well-characterised *Salmonella* strain 4/74. The ability to utilise lactose was a property of most *S*. *diarizonae* isolates, consistent with previous reports that 85% of *S*. *diarizonae* are Lac^+^ [82]. It is estimated that less than 1% of all *Salmonella* ferment lactose due to the loss of the *lac* operon from the *S*. *enterica* subspecies [83]. It was interesting to discover that one non-venomous snake isolate (13L-2837) which belongs to *S*. *enterica* clade B was capable of utilising lactose as a sole carbon source. Isolate 13L-2337 belongs to the serovar *S*. Johannesburg and to our knowledge this is the first published occurrence of a Lac^+^ *S*. Johannesburg isolate. The 13L-2837 pan-genome had zero percent homology to the *lac* genes from reference strain *E*. *coli* MG1655 (sequence in S1 Text) (results in Fig 3), suggesting an alternative method for lactose utilisation. The 13L-2837 *S*. Johannesburg isolate also lacked the ability to grow on dulcitol, despite possessing all of the relevant *gat* genes, raising the possibility of an inverse relationship between the ability of *Salmonella* to utilise dulcitol and lactose as a sole carbon source. These findings require further investigation which is beyond the scope of the current study.

The majority of *S*. *enterica* clade A and clade B isolates utilised dulcitol, whereas dulcitol was rarely used as a sole carbon source by *S*. *diarizonae*. These findings are consistent with a study of *Salmonella* derived from Australian sleepy lizards, which demonstrated that dulcitol utilisation was observed in almost all *S*. *enterica* and *S*. *salamae* isolates but only 10% of *S*. *diarizonae* isolates [84]. Over 50% of the *S*. *enterica* clade A isolates lacked the *gatY* gene but grew well on dulcitol as a sole carbon source, suggesting that GatY is not required for dulcitol catabolism. A variety of repertoires of dulcitol catabolic genes have been described across *Salmonella*, with individual serovars carrying one of two *gat* gene clusters [59]. Both of these clusters carry the *gatY* gene. Our findings may indicate that a third *gat* gene cluster is carried by some *Salmonella* serovars.

In the majority of cases, allantoin was only utilised as a sole carbon source by *S*. *enterica* clade A isolates, consistent with a previous report that described an association of clade A with the allantoin catabolism island [9]. The majority of clade B isolates lacked the allantoin catabolism island and thus were unable to utilise allantoin as a sole carbon source. However, we identified one clade B isolate as an exception, isolate 11L-2351 which was sampled from a non-venomous reptile. This isolate belongs to the serovar Montevideo, which is frequently associated with outbreaks of human salmonellosis [85–87]. In reptiles, the end product of the purine catabolic pathway is not allantoin, but uric acid [9]. The consequent absence of allantoin from the snake gastrointestinal tract could explain why a substantial number of *S*. *enterica* clade B were found in snakes.

It is possible that the gain and loss of allantoin catabolic genes is relevant to host specificity. A relationship between the pseudogenization of the allantoin metabolic genes and niche adaptation has also been proposed for the invasive nontyphoidal *Salmonella* (iNT*S*) reference isolate for *S*. Typhimurium: D23580 [49,88]. Compared with *S*. Typhimurium isolate 4/74, which shows a broad host range, D23580 is unable to utilise allantoin as a sole carbon source, consistent with the adaptation of invasive *Salmonella* in Africa towards non-allantoin producing hosts [49,88]. Furthermore, accumulation of pseudogenes in the allantoin degradation pathway has been reported in host-restricted *Salmonella* serovars which cause invasive disease, suggesting that the ability to grow on allantoin is a marker of a switch from enteric to invasive disease [89]. These findings may reflect the clinical observation that snake-acquired salmonellosis is frequently an invasive disease that commonly results in hospitalisation, compared to disease caused by *Salmonella* derived from allantoin-producing hosts.

### Perspective

Although reptiles are known to harbour a diverse range of *Salmonella* bacteria, until now *Salmonella* carriage has not been examined in many key reptilian species. Here, we have shown that venomous snakes harbour and shed a wide variety of *Salmonella* serovars that represent much of the spectrum of the *Salmonella* genus and are phylogenetically distributed in a similar way to *Salmonella* found in non-venomous reptiles. We demonstrated that venomous snakes can carry and excrete *Salmonella* serovars which cause human disease. One of the *Salmonella* isolates was resistant to first-line antimicrobial agents. It is possible that venomous snakes represent a previously uncharacterised reservoir for *Salmonella* both in captive settings and in the wider environment. Further study is required to investigate the relationship between clinical cases and reptile-derived *Salmonella* in tropical regions inhabited by venomous reptiles such as Africa. We believe that our study provides a good baseline for this future work.

Reptiles are an ideal population of animals for the study of genus-level evolution of *Salmonella* because they carry phylogenetically diverse isolates that belong to the majority of *Salmonella* subspecies. By demonstrating the phenotypic and genotypic conservation of metabolic properties across three phylogenetic groups of *Salmonella* we have shed new light on the evolution of *Salmonella* serotypes.

## Supporting information

S1 Table Reptile-associated-*Salmonella* strain metadata and control strain metadata. The table includes all metadata associated with the venomous snake, non-venomous reptile and control strains used throughout this research including details on accessing raw reads where applicable.
(XLSX)

S2 Table Contextual reference genomes metadata and accession numbers. The table lists the strains used in phylogenetic analysis to put the study strains into wider context and includes some basic information in addition to accession numbers.
(XLSX)

S3 Table QUAST assembly statistics. The table provides details on assembly statistics for each sequenced genome and highlights those strains which failed quality control in red.
(XLSX)

S4 Table Carbon source utilisation gene information. The table provides a list of the carbon utilisation genes together with rationale for why each gene was chosen. A list of the carbon source, gene name and gene product is provided along with the LT2 Locus ID (STM number), the SL1344 Locus ID (SL1344 number), a link to the KEGG pathway and a citation demonstrating the activity of the gene in *Salmonella*.
(XLSX)

S5 Table Carbon source specific requirements. The table contains a list of the carbon sources used to determine the metabolic capacity of reptile-associated *Salmonella*. Specific conditions, concentrations and references are given for each.
(XLSX)

S1 Text Carbon source utilisation gene sequences. A full list of the sequences used to determine the presence or absence of genes involved in carbon source utilisation in reptile associated *Salmonella*.
(FASTA)

S1 Fig The diversity of *Salmonella* isolated from a collection of venomous snakes and non-venomous reptiles including the *S.* bongori outgroup. Core genome maximum likelihood phylogenetic tree. The tree was rooted using *S*. *bongori* shown here. 25 contextual reference genomes representing previously sequenced isolates from each *Salmonella* subgroup are indicated in purple.
(TIF)
