# Supplementary figures and images for: The diversity, evolution and ecology of Salmonella in venomous snakes
Source: PLoS Negl Trop Dis. 2019 Jun 4;13(6):e0007169. doi: 10.1371/journal.pntd.0007169 (PMC6548357; doi:10.1371/journal.pntd.0007169)

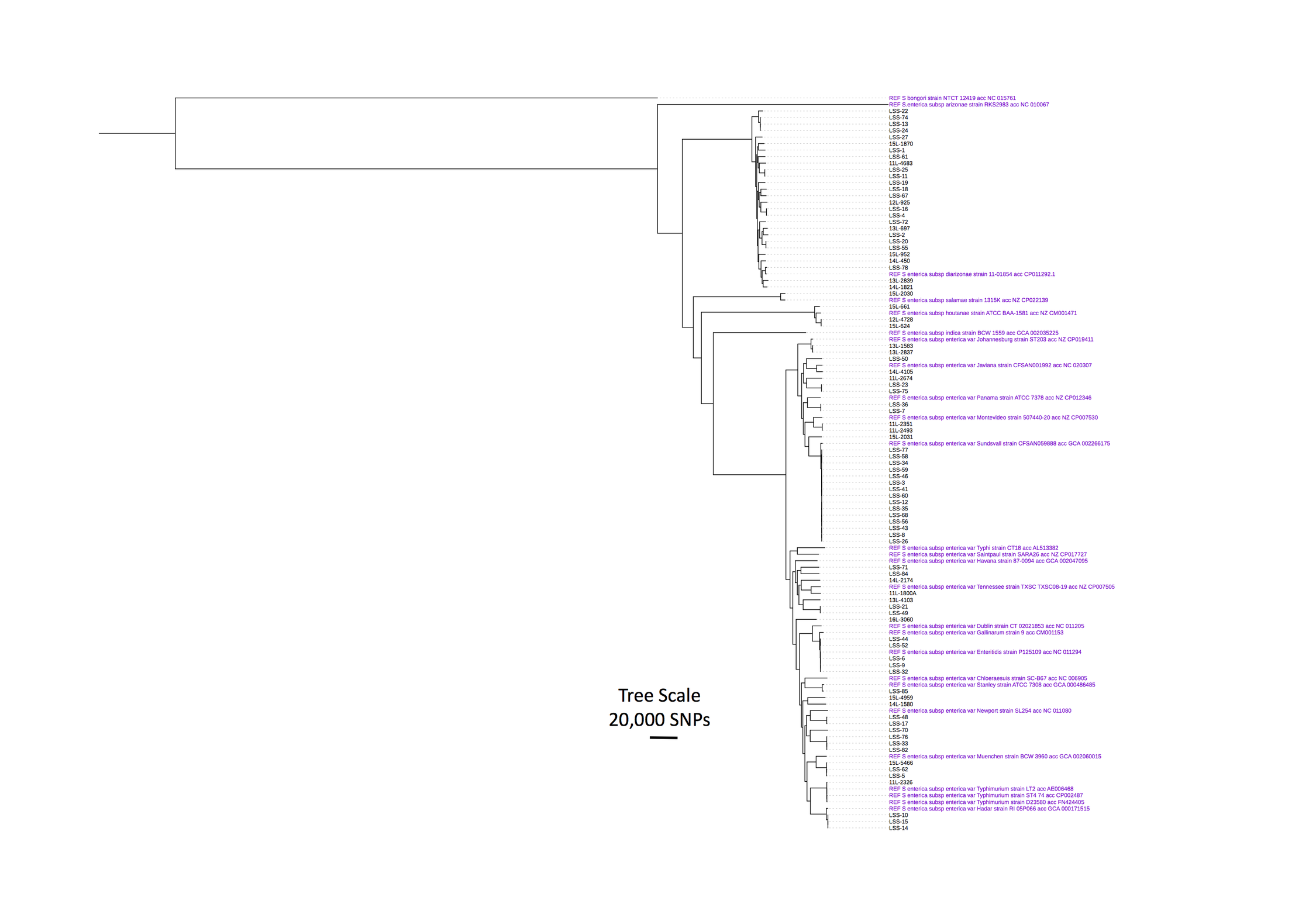

Supplement: S1 Fig — Core genome maximum likelihood phylogenetic tree. The tree was rooted using S. bongori shown here. 25 contextual reference genomes representing previously sequenced isolates from each Salmonella subgroup are indicated in purple. (TIF) [file pntd.0007169.s007.tif]
